# Patient advocacy groups: Need and opportunity in India

**DOI:** 10.4103/2229-3485.76283

**Published:** 2011

**Authors:** Kunal Shah, Sunil Garg

**Affiliations:** *Eisai Clinical Research Singapore Pvt. Ltd., Mumbai, India*; 1 *INC GVK BIO Pvt. Ltd., Gurgaon, India*

**Keywords:** Healthcare providers, government, media, patient advocacy, research

## Abstract

With an increasing number of corporate hospitals, healthcare related issues, research trials and undue attention by media in India, there is a need to focus more on patient’s rights and protection. In India, multiple agencies like regulatory bodies, scientific review committees, ethics committees, NGOs, etc. work toward patient rights and protection. However, these agencies are inadequate to cater to the general issues related to patient’s rights. There’s a need to have a separate group of people who provide advocacy to the patient, or simply, a patient advocacy group which will work explicitly in these areas to increase transparency and credibility of healthcare system in India. This group will provide special attention to patient care and protection of rights from the planning stage rather than at the troubleshooting stage.

## INTRODUCTION

Advocacy means “set of actions whose main objective is to sensitize with a view to influencing decisions about a cause or policy in a stated direction” and is done by “pleading or arguing in favor of something”. In social advocacy, the people are the objects of the stated good to arise from such actions. In patient advocacy, patients are the objects of the stated good to arise from such actions. Advocate is a person who does the act of pleading or arguing in favor of something.

Patient advocate is a liaison between patients and healthcare providers (HCPs) in order to improve or maintain high quality of healthcare for the patients. The terms “patient advocate” and “patient advocacy” have a broad range of usage and may be applied to the actions of many different individuals and organizations.[1]

## TYPES OF PATIENT ADVOCATES

### Lay patient advocate

May be a patient himself/herself who is conscious and reasonably aware of the circumstances or a relative or friend or someone else who is personally concerned about the patient’s welfare.

### Professional individual patient advocate

These are individuals, organizations and agencies that offer individual patient advocacy services for a fee.

### Governmental agencies

These are central or state agencies that oversee HCPs. These offices are responsible for certifying and recertifying healthcare professionals and healthcare institutions.[1] They issue reports which can be helpful to patients on all providers within their area of oversight.

### Patient advocacy within the healthcare community

Some hospitals, health insurance companies, and other healthcare organizations employ people specifically to assume this role. Within hospitals, the person may have the title Patient Representative.[1]

### Nursing and patient advocacy

Advocacy in nursing finds its theoretical basis in nursing ethics. The nurse’s primary commitment is to the patient, whether an individual, family, group, or community. The nurse has responsibility to promote, advocate for, and strive to protect the health, safety, and rights of the patient.[1]

### Patient advocacy groups

There are the organizations, generally non-profit, that focus on specific diseases or aspects of healthcare. These are sometimes distinguished from Patient Advocates and referred to as Health Advocacy Organizations,[1] e.g. Alagille Syndrome Alliance, CARES Foundation, CSS Patient Group, Komen Advocacy Alliance.

All the above types of patient advocacy, except governmental agencies and PAGs, cater to individual patient or a small group of patients in a specific area or a hospital but are not able to do all the requisite tasks needed.

## ROLE OF PATIENT ADVOCACY GROUPS IN INDIA

Though we have governmental agencies and NGOs who are performing many activities in healthcare sector, there is no single organization in the country which will truly qualify as a PAG. Such groups can further improve the overall quality of healthcare system in India by supporting the patients in fighting for their rights, welfare and safety with HCPs. PAGs can play an important role in improving the quality of healthcare system in India by the following means.

1. Communication and collaboration with patient and their families:

- To help patient/patient’s caregiver understand about the disease the patient has and the alternative therapies available.[1]
- To help the patient achieve higher percentage of successful treatment and compliance by providing continuous guidance to the patient/patient’s caregiver about the tests and procedures the patient might undergo, by training on routine homecare and importance of the adherence to the treatment schedule.
- To help the patients manage their finance by assisting them with insurance and household accounting management. Sometimes, health insurance and other cost and billing issues can be very confusing; therefore, patient advocates can help patients or their families to wade through the confusion.[1]
- To generate funds for non-affording patients from various sources like NGOs, government institutions, trusts, donors, etc. for appropriate treatment.

2. Support healthcare research:

- To create awareness and promote research activity with ethics and science.
- To get access to patient’s and/or clinical trial registries/database and facilitate clinical trial participation.
- To communicate research results to the society in a more comprehendible way.

3. Communication and collaboration with government agencies:

- To promote healthcare system that is realistic for patients and HCPs and not merely beneficial to corporations.[1]
- To provide assistance for development and review for research protocol, informed consent document, etc. through their medical and scientific advisory boards.
- To ensure that ongoing projects and those being considered for funding by government directly impacts patient’s lives, improving delivery of care and support by working on investigative and advisory panels.[1]
- To work with finance boards, analyze cost constraints and act as a proponent for best practices, and advocating better protection for patients and HCPs.[1]
- To improve the quality of healthcare by influencing the government to modify the existing policies or design new policies in favor of patients and other stake holders.

4. Communication with media:

- To sensitize media members regarding the practical aspect of healthcare and research related activities.
- To identify the root cause and plan corrective action for conflicting research issues rather than blowing issues out of the proportion, by acting as a facilitator between practitioner, corporate and media.

## OPPORTUNITY FOR VARIOUS STAKE HOLDERS IN INDIA

Considering the scope of work for PAGs and the current environment in India, the stake holders needs to look at the opportunities mentioned below instead of perceiving it as a threat.

- HCPs can get helping hand for conveying the right treatment to patients and families, get more compliance.
- Patients can take more informed decision in addition to getting access to new treatments and cost benefits.
- Pharma companies can create more transparency about their deeds and acts and also get indirect help to promote research activities.
- Government agencies can get additional manpower to learn about perceptions and needs of different stake holders and take balanced decisions in policy making, in development and review of research documents, etc.
- Students can see this as an opportunity to pursue this field as a career option.
- Educational institutions can see this as an opportunity to design specific courses to supply talent pool for this upcoming field.

## SERVICES PATIENT ADVOCATE CAN OFFER[2]

Patient advocate can offer many types of services for patients which include the following.

### Medical assistance

They help review diagnoses, treatment options, medical records and test reports, may accompany a patient to appointments, monitor the patient at the bedside in a hospital or be a good choice for a healthcare proxy.

### Insurance assistance

They help to choose the best insurance plan, handling paperwork and insurance filings, negotiating denials of claims, and Medicare plan decision-making.

### Home health assistance

Home health assistants may or may not have any nursing-type training. They can perform services such as in-home nursing care, home therapy and rehabilitation, and daily living assistance.

### Elder and geriatric assistance

This type of assistance may also be found in assisted-living and nursing home settings, and may provide help with Medicare services or hospice services.

### Legal assistance

Sometimes, the help needed for medical problems is really more legal in nature. From worker’s compensation to disability filings, to malpractice and medical error review, the lawyer is the best advocate.

## BECOMING A PATIENT ADVOCATE

The advocates and care managers providing this type of care have, in most cases, a medical background, i.e. retired doctors or nurses, or someone who spent the first part of his career in a medical or paramedical capacity. In addition, the person will have to understand the local legal framework and policies.

Nine areas of practice and knowledge are identified as crucial for the position of a patient and consumer advocate by The Society for Healthcare Consumer Advocacy of the American Hospital Association, which are patient rights, grievance and complaint management, patient satisfaction measurement, customer service, mediation/conflict negotiation, crisis intervention, data management and healthcare management.[3]

Outside India, there are universities and groups which run programs and certificate courses for patient advocacy, e.g. Cleveland State University runs online program specifically for advocates who work in hospitals; a group called Healthcare Liaison located in Berkeley, California, runs workshops to teach people to be private healthcare advocates; The University of Connecticut Health Center offers Health Ecademy, an online course; The University of Miami (Florida) offers the Alfus Patient Advocacy Certificate online; The University of Toledo (Ohio) offers a two-semester Patient Advocacy Online Graduate Certificate Program; Sonoma (California) State University offers a two-semester program in Patient Advocacy and Navigation training with a special emphasis on Integrative Health Care; Sarah Lawrence College offers Masters Degree in Health Advocacy (the oldest formal health advocacy program in the United States), to name a few.[4] The same trend may be followed after increasing the awareness and improving the legal framework in India.

So, in a nutshell, PAGs are not the ones who only look at ethics part and do not consider practical and scientific issues or only come into picture at the time of controversy or issues or are against research or pharma companies or only criticize either a pharma company or HCP but also they work with them to understand and improve the situation. Rather, they are the ones who perform advocacy at every level for all patients and families to protect their rights by either communication with media and government as a concerned citizen or lobbyist or by promoting research to get access to newer more effective medicines or by communication with patient and families to assist them in daily activities and healthcare procedures and select the best treatment option and make fully informed decisions.[1]

## DISCLAIMER

All opinions expressed herewith are those of the authors and not of any of the organizations.
